# Could *Chlorella pyrenoidosa* be exploited as an alternative nutrition source in aquaculture feed? A study on the nutritional values and anti-nutritional factors

**DOI:** 10.3389/fnut.2022.1069760

**Published:** 2022-12-07

**Authors:** Fufeng Chen, Jun Qian, Yu He, Yunyue Leng, Wenguang Zhou

**Affiliations:** ^1^Key Laboratory of Poyang Lake Environment and Resource Utilization, Ministry of Education, School of Resources and Environment, Nanchang University, Nanchang, China; ^2^Xinjiang Rao River Hydrological and Water Resources Monitoring Center, Shangrao, China

**Keywords:** microalgae, nutrition, aquaculture, anti-nutritional factor, protein

## Abstract

This work attempted to identify if microalgal biomass can be utilized as an alternative nutrition source in aquaculture feed by analyzing its nutritional value and the anti-nutritional factors (ANFs). The results showed that *Chlorella pyrenoidosa* contained high-value nutrients, including essential amino acids and unsaturated fatty acids. The protein content in *C. pyrenoidosa* reached 52.4%, suggesting that microalgal biomass can be a good protein source for aquatic animals. We also discovered that *C. pyrenoidosa* contained some ANFs, including saponin, phytic acid, and tannins, which may negatively impact fish productivity. The high-molecular-weight proteins in microalgae may not be effectively digested by aquatic animals. Therefore, based on the findings of this study, proper measures should be taken to pretreat microalgal biomass to improve the nutritional value of a microalgae-based fish diet.

## Introduction

Aquaculture, which provides meat products to humans, is an important economic sector worldwide (1). To support the development of the aquaculture industry, continuous input of high-quality animal feed is necessitated. In the past, due to its high digestibility and high nutritional value, fishmeal was widely added to the feed to provide proteins to aquatic animals. However, in recent years, with the gradual depletion of offshore fishery resources, the production of high-quality fishmeal is negatively impacted and the cost of fishmeal is on the rise. Under this circumstance, researchers are trying to explore new protein resources for the aquaculture industry (2).

Notably, soybean meal is a good alternative protein resource for animal rearing. In the real-world application, to improve the digestibility of soybean meal, enzymatic treatment is usually adopted. Haghbayan and Mehrgan (3) reported that enzyme-treated soybean meal (ESBM), as a protein source, could be added to the animal feed. In addition to soybean meal, microalgal biomass is regarded as a potential protein resource in the feed production industry. The protein content of some microalgal species, including *Chlorella* sp. and *Scenedesmus* sp., reach over 50% of the total biomass (dry weight) (4, 5). Compared to soybean cultivation, microalgae production has advantages in terms of productivity and land utilization. First, if the depth of the algae production system, one-batch duration, and maximum biomass density (dry weight) are set to 0.5 m, 15 days, and 1.2 g L^−1^, respectively, the biomass yield of microalgae grown on 1,000 m^2^ land in 3 months is 3,600 kg. However, the maximum biomass yield of soybean cultivated on 1,000 m^2^ of land in 3 months is only 350 kg. Second, microalgae production is possible in ocean and desert ecosystems, with no competition from traditional agricultural crops, while soybean cultivation on a large scale needs large fertile lands (6). Therefore, the utilization of protein-rich microalgae for feed production and animal rearing is attracting research attention.

In the past, researchers from academia and industry devoted several efforts to technological innovations for the industrial use of microalgae biomass. To date, microalgae production bioreactors, biomass collection devices, and low-cost drying techniques have been industrialized (7, 8). Recently, evidence evaluating the effects of feed supplemented with microalgae on the growth rate and meat quality of animals is accumulating. For example, Deng et al. (4) replaced 5–20% of fishmeal in a fish diet with *Chlorella* sp. to support the growth of *Micropterus salmoides* (4). This is a decisive step from the research environment into the industry. To our knowledge, three important questions should be addressed before the industrial use of microalgal biomass in animal feed. First, does the microalgal biomass contain essential nutrients for animal rearing? Second, are there any anti-nutritional factors (ANFs) in microalgal biomass limiting its usage in animal feed? Third, what are the critical control points for using microalgal biomass as an alternative resource for fish rearing?

This study primarily aimed to explain the feasibility of using microalgal biomass as an alternative resource for fish rearing in practice based on the analysis of the corresponding nutritional value. Nutrition-related parameters mainly included the contents of proteins, lipids, carbohydrates, essential elements (P, Ca, Mg, K, etc.), fatty acids and amino acids, ANFs, and the molecular characterization of proteins. According to the experimental results, technologies that can promote the advancement of microalgae-based animal feed toward industrialization are discussed. It is expected these findings are beneficial to the industrialization of microalgal biomass as an alternative resource for fish rearing.

## Materials and methods

### Microalgae, fishmeal, and soybean meal

*Chlorella pyrenoidosa*, an algal strain with high protein content, was cultivated in a TAP medium for 6 days. The medium composition and cultivation conditions are documented in our previous publication (9). By the end of the microalgal cultivation, biomass was collected by centrifugation and dehydrated using a vacuum dryer (Alpha 1-4 LDplus, Christ, Osterode, Germany). Fishmeal and ESBM were obtained from Tongwei Co. Ltd and Hamlet Protein Inc., respectively. ESBM is the defatted soybean treated by specific enzymes. Fishmeal, ESBM, and dehydrated microalgal biomass were sealed and stored in a refrigerator (4°C) until the experiment. Samples of microalgal biomass, ESBM, and fishmeal for the experiment are shown in Supplementary Figure S1.

### Experimental design

This study was conducted in three steps to evaluate if microalgal biomass could serve as an alternative nutrition source in aquaculture feed. First, nutritional values of microalgal biomass were assessed by measuring the contents of proteins, lipids, some essential elements, amino acid profile, and fatty acid profile. Nutritional values of the microalgal biomass, ESBM, and fishmeal were compared. Second, major ANFs, including trypsin inhibitor activity, α-amylase inhibitory activity, total saponin content, lectin, tannins, and phytic acid, in microalgal biomass and ESBM were quantified. Third, molecular weight distributions of proteins in microalgal biomass, ESBM, and fishmeal were obtained. In this study, all the experiments and tests were conducted in triplicates. The results were expressed as mean ± standard deviation.

### Analytical methods

#### Analysis of nutritional components

The protein content was measured by the Kjeldahl (KN580, ALVA, China) method (10). The lipid content was obtained by repeated extraction with chloroform-methanol (2:1 v/v) and weighing the evaporated solvent. Fatty acids were analyzed using GC-MS (GC-MS QP2010SE, Shimadzu) based on the peak area for identification and relative quantitative analysis. Details of the analysis of lipids and fatty acids were described previously (11). The total carbohydrate content was determined based on the method described by Chow and Landhäusser (12). The samples were first subjected to chloroform-methanol (2:1 v/v) isolation, followed by a phenol-concentrated sulfuric acid reaction, and glucose was used as the standard solution to measure the absorbance at 490 nm using a spectrophotometer (DR 6000, Hach, Loveland, USA). Moisture was measured using a digital balance (0.0001 g) after drying in the oven at 105°C to constant weight. Ash content was determined by burning the sample in a muffle furnace at 550°C. The crude fiber content was determined based on the Chinese National Standard GB/T 5009.10-2003. The principle was as follows: under the action of sulfuric acid, the sugar, starch, pectin, and hemicellulose in the sample are hydrolyzed and removed. Subsequently, treatment with alkali removes proteins and fatty acids, and the remaining residue is crude fiber. By accurately weighing a 0.05 g sample, dissolved in 5 mL ultrapure water, and shaking for 2 min, the water-soluble pH was determined after 1 and 2 h.

The samples were accurately weighed to 50 mg (triplicate), extracted by adding 5 mL 80% ethanol, centrifuged for 10 min (10,000 rpm at 20°C) after incubation for 20 min in a water bath (80°C), and the supernatant was combined once again and used to determine total phenolic and flavone contents; the residue was dried to determine amylum levels. Total phenolics and flavone were determined following the method described by De França Silva et al. (13) with minor modifications. Briefly, total phenolics were determined by the Folin-Denis method, whereby 800 μL of the diluted extract was added to 4.2 mL of pure water and 0.2 mL of Folin-Denis reagent. The solution was thoroughly mixed and made to react at room temperature for 3 min. Subsequently, 20% Na_2_CO_3_ solution (0.4 mL) was added, and the mixture was evenly mixed and left to stand for 30 min. The absorbance was measured at 760 nm on a spectrophotometer (DR 6000, Hach, Loveland, USA). The total phenolics concentration of the sample was calculated by comparing the absorbance of the extract using the standard curve for tannins, expressed in mg tannin equivalents per gram dry weight of the sample. The determination of flavone was consistent with the protocol described by De França Silva et al. (13) and was expressed in mg rutin equivalent per gram dry weight of the sample. Following the procedure in Fernandes et al. (14), amylum was determined using the perchloric acid method. Specifically, the residue was dried and extracted using 2 mL 30% HClO_4_ for 90 min and centrifuged for 20 min (10,000 rpm at 4°C). The diluted supernatant (2 mL) was extracted and made to react with the iodine reagent (1 mL) in a glass test tube for 10 min. The absorbance of the mixture was measured at 620 nm (DR 6000, Hach, Loveland, USA). The amylum concentration in the sample was calculated using starch as the standard.

The determination of elemental analysis (phosphorus, calcium, magnesium, potassium, and sodium) was based on the Chinese National Standard GB/T 35871-2018. Briefly, the intensity of the sample was measured using ICP-MS (Agilent 7700S) after microwave digestion, and its elemental content was calculated based on a standard curve.

The amino acid detection was performed in the Shiyanjia Lab (www.shiyanjia.com), and the specific protocol was based on the Chinese national standard GB/T 18246-2019. The samples were hydrolyzed and the contents of 17 amino acids were analyzed using an automatic amino acid analyzer (Hitachi L8900, Japan).

#### Analysis of ANFs

Trypsin inhibitor activity (TIA) was determined by an enzymolysis reaction following the protocol described in Shang et al. (15). TIA was expressed as mg of trypsin inhibition per gram of sample. The determination of lectin content was performed following the method described by Chu et al. (16); ethanol was selected as the extract, and sheep blood was selected as the agglutination object. First, a certain amount of sample was dissolved in 20% ethanol solution at a concentration of 1:10 (g/mL), mixed, and incubated at 4°C for 12 h, followed by centrifugation for 20 min (8,000 rpm at 4°C). The supernatant was collected as the extract. Freshly obtained sheep blood was centrifuged for 10 min (5,000 rpm at 4°C); the supernatant was removed, washed thrice with PBS buffer, and dissolved in 2% PBS solution. PBS buffer (100 μL) was added to the wells of a 96-well V hemagglutination plate, and 50 μL of the extract was added to the first well and diluted several times. Red blood cell suspension (100 μL) was added to each well, and the titer was determined after incubation at room temperature for 2 h. The inverse of the highest two-fold dilution showing positive hemagglutination was the extract titer.

The total saponin content was determined using oleanolic acid as the reference, and the specific protocol described by Shang et al. (15) was followed. The total saponin content in this study was expressed in oleanolic acid glycosides (17, 18). The inhibitory activity of amylase was analyzed by measuring the inhibition efficiency of the samples. The α-amylase inhibitory activity was measured by the di-nitro-salicylic acid (DNS) method (19). The sample was accurately weighed to 50 mg, added to a 10 mL sodium acetate (0.05 mol L^−1^, pH = 7.0) solution, and stirred at 20°C for 12 h, followed by centrifugation for 30 min (8,000 rpm at 4°C). The supernatant (2 mL) was accurately measured; subsequently, 0.4 mL of the soluble starch solution (2%), 0.2 mL of the amylase solution (2.0 g L^−1^), and 0.2 mL of citrate buffer (0.1 mol L^−1^, pH = 5.6) were added, and thoroughly mixed. After the mixture was incubated at 37°C in a water bath for 30 min, the DNS reagent (3.0 mL) was added and mixed in a water bath at boiling temperature for 10 min. After cooling to room temperature, the reaction solution (0.5 mL) was measured; 4.5 mL of water was added, and the mixture was mixed. The absorbance, denoted as OD_1_, was measured at 520 nm (DR 6000, Hach, Loveland, USA). Water was used instead of the α-amylase inhibitor, and its absorbance was denoted as OD_2_. The blank was distilled water, and its absorbance was denoted as OD_3_. The inhibition rate of α-amylase was calculated as follows: AU = (OD_2_-OD_1_)/ (OD_2_-OD_3_) × 100%.

Phytic acid content was determined according to national standard GB 5009.153-2016. The sample was extracted using an acidic solution and desorbed on an anion exchange resin. The phytic acid in the eluent was made to react with sulphosalicylic acid-ferric chloride. The absorbance was measured at 500 nm on a spectrophotometer, and the phytic acid content was calculated using sodium phytate as the standard. Tannin content was determined according to the Chinese national standard GB/T 27985-2011. The tannins in samples were extracted using acetone solution, centrifuged for 10 min (8,000 rpm at 4°C), and the supernatant was added into the mixture of sodium tungstate-phosphomolybdic acid and sodium carbonate solutions. The absorbance was measured at 760 nm, and tannic acid was used as the standard to calculate the content of tannins in the sample.

#### Analysis of the molecular distribution of proteins

Relative molecular distributions of proteins were determined in the Shiyanjia Lab (www.shiyanjia.com). The proteins in the samples were extracted and lyophilized, and 40 μL of the trypsin buffer was added and incubated for 16–18 h at 37°C for enzymolysis. The enzymolytic products were separated by capillary high-performance liquid chromatography and analyzed on a Q Exactive mass spectrometer (Thermo Fisher). The analysis time was 60 min. The positive ion detection method was used. The mass charge ratios of peptides and fragments of peptides were collected as follows: ten fragment profiles were collected after each full scan. MaxQuant 1.5.5.1 was used to search the corresponding database for the original documents of mass spectrometry testing, and the results of protein identification and quantitative analysis were obtained.

## Results

### Analysis of nutritional values

#### Quantification of the nutritional components

The *C. pyrenoidosa* biomass comprised high protein (52.4%) and lipid (19.4%) contents (Table 1), which are essential to the growth of aquatic animals. Compared to ESBM, *C. pyrenoidosa* contained higher proteins and lipids. However, the protein content of *C. pyrenoidosa* biomass was much lower than that (62.9%) of the fishmeal. Previous studies have developed several strategies to improve the protein content in microalgae. For example, Lu et al. (20) applied zeolite as a slow-releaser of ammonia in the culture medium for *Spirulina* sp. and showed that the slow-release of ammonia enhanced the protein accumulation in *Spirulina* sp., increasing its content to 69.8%.

**Table 1:** Nutritional analysis for fishmeal, microalgae, and enzyme-treated soybean meal (ESBM).

	**Fishmeal**	**Microalgae**	**ESBM**
Crude protein (%)	62.91 ± 0.54	52.36 ± 0.64	52.17 ± 0.29
Lipid (%)	11.13 ± 0.28	19.36 ± 1.04	4.02 ± 0.48
Polysaccharide (%)	0.54 ± 0.01	9.01 ± 0.43	9.00 ± 0.11
Moisture (%)	9.12 ± 0.58	6.45 ± 0.14	4.47 ± 0.25
Ash content (%)	15.96 ± 0.14	6.93 ± 0.01	7.32 ± 0.05
Other components (%)	0.34	5.89	23.02
Crude fiber (%)	/	3.38 ± 0.10	6.16 ± 0.06
pH of dissolved water			
(1 h)	6.65 ± 0.01	7.10 ± 0.01	6.68 ± 0.04
(2 h)	6.65 ± 0.02	6.99 ± 0.01	6.54 ± 0.02
Amylum (mg g^−1^)	/	6.63 ± 0.22	6.37 ± 0.23
Flavone (mg g^−1^)	/	37.11 ± 1.47	0.96 ± 0.01
Total phenolics (mg g^−1^)	/	6.82 ± 0.17	5.06 ± 0.11

Microalgal biomass contains flavones and phenolics, the bioactive and immune-enhancing components in animal culture (21, 22). Notably, the contents of flavones and phenolics in microalgal biomass were 37.1 and 6.8% higher than those in ESBM, respectively. Hence, microalgae added to the fish diet can serve as a source of bioactive components to promote the growth of aquatic animals. Microalgae also contain some components which may be unfavorable to fish growth. Microalgae contained some amylum (Table 1), which may not be digested efficiently by carnivorous fish.

Supplementary Table S1 indicates that microalgae biomass contains 0.36% magnesium (Mg) and 1.25% potassium (K), slightly higher than the corresponding contents in the fishmeal. However, the content of calcium (Ca) in microalgae was much lower than that in fishmeal. Simultaneously, the ratio of Ca/P in fishmeal reached 1.30 while that in microalgae was only 0.14. This may be attributed to the fact that fishmeal contains a high portion of fish bones, enriched in Ca. Therefore, in a real-world application, Ca element can be supplemented in an algae-based fish diet to prevent its deficiency.

#### Amino acid composition

*C. pyrenoidosa* contain several amino acids, including essential amino acids (EAAs) and non-essential amino acids (NEAAs) (Table 2). The amino acid content of *C. pyrenoidosa* was 189.7 mg g^−1^, which was consistent with the content of *C. pyrenoidosa* (186.6 mg g^−1^) in the study of Zhong et al. (23). However, the amino acid content of microalgae depended on different culture conditions, such as medium composition (24–26), temperature (27), stress stimulation (23, 28, 29) and other factors (30). For example, Zhong et al. (23) found that adding 20–80 mg L^−1^ selenium (Se) could promote amino acid accumulation in *C. pyrenoidosa* biomass, and the addition of 40 mg L^−1^ Se achieved the highest amino acid accumulation (237.1 mg g^−1^) among the treatments. Although the content of total amino acids (189.7 mg g^−1^) in *C. pyrenoidosa* was lower than that (226.9 mg g^−1^) in the fishmeal, *C. pyrenoidosa* showed a generally-balanced amino acid composition. Therefore, *C. pyrenoidosa* biomass is a high-quality protein source for aquaculture.

**Table 2:** Amino acid composition of fishmeal, microalgae, and enzyme-treated soybean meal (ESBM).

**Amino Acids (AA)** **(mg g^−1^)**	**Fishmeal**	**Microalgae**	**ESBM**
**EAA** ^a^			
Arginine	28.273	27.865	27.987
Histidine	6.961	3.606	4.656
Isoleucine	9.606	6.957	8.943
Leucine	19.844	17.828	17.741
Lysine	16.461	10.269	9.898
Methionine	8.902	5.520	3.850
Threonine	9.368	7.369	7.294
Phenylalanine	10.105	13.284	10.153
Valine	17.887	15.815	15.093
Cystine	0.181	0.108	0.100
**Total**	127.588	108.621	105.715
**NEAA** ^b^			
Alanine	13.140	12.842	8.283
Aspartate	18.503	13.864	18.310
Glutamate	27.222	19.654	29.581
Glycine	11.996	8.700	7.681
Proline	10.542	9.838	9.299
Serine	9.339	7.683	9.718
Tyrosine	8.555	8.502	8.754
***Total***	99.297	81.083	91.626
**Total AA** ^c^	226.885	189.704	197.341

a EAA, essential amino acid.

b NEAA, non-essential amino acid.

c AA, amino acid.

#### Fatty acid composition

Fatty acid compositions of the three samples are shown in Supplementary Table S2. Comparison between microalgae and ESBM demonstrated that microalgae contained a substantially higher percentage of unsaturated fatty acids than the soybean meal. The percentage of unsaturated fatty acids in microalgae was higher relative to the fishmeal. Previous studies suggest that unsaturated fatty acids in microalgae biomass are favorable to the growth and immune responses of aquatic animals (31–33). Therefore, the replacement of fishmeal or soybean meal by microalgae in aquaculture practice can improve the percentage of unsaturated fatty acids in the lipid profile.

However, the percentage of polyunsaturated fatty acids (PUFAs) in microalgae used in this study was very low (1.2%). To our knowledge, some microalgal species, like *Chlorella* sp., can be a good source of PUFAs (34). The main reason for the low percentage of PUFAs in microalgae in this study may be attributed to the conditions of cultivation of microalgae, wherein no inducing factor was applied. Therefore, in a real-world application, to improve the nutritional value of a fish diet supplemented with microalgae, inducing conditions should be adopted to improve the percentage of PUFAs in microalgae.

### Analysis of ANFs

When microalgae and soybean meal are added to a fish diet, it is important to consider ANFs. ANFs may impair nutrient utilization, thus interfering with fish performance and health (35, 36). Hence, the contents of ANFs in fishmeal substitutes should be accounted for. Both microalgae biomass and soybean meal contained some ANFs, including trypsin inhibitor activity, saponin, lectin, phytic acid, and tannins (Table 3). Previous studies suggest that the aforementioned ANFs can negatively impact the performance and health of aquatic animals *via* different mechanisms (37). For example, tannins in the fish diet can interfere with digestion by inactivating digestive enzymes and form strong complexes with protein and minerals in the feed (38, 39). Omnes et al. (40) discovered that in the culture of juvenile European seabass, a 10 g kg^−1^ tannin-containing diet significantly lowered the protein digestibility (40). In the culture of turbot, higher levels of phytic acid in the diet adversely affected nutrient utilization by the fish (41). Therefore, to add microalgae to the fish diet as a nutrition source, the contents of ANFs in microalgal biomass should be considered.

**Table 3:** Analysis of anti-nutritional factors (ANF) in microalgae and enzyme-treated soybean meal (ESBM).

	**Microalgae**	**ESBM**
Trypsin inhibitor activity (TIA: mg g^−1^)	0.52 ± 0.04	0.80 ± 0.02
Lectin content^a^	2^4^	2^1^
Total saponin content (mg g^−1^)	40.68 ± 0.53	13.05 ± 1.91
α-amylase inhibitory activity (%)	-	-
Phytic acid (mg g^−1^)	0.62 ± 0.10	17.13 ± 0.41
Tannins (mg g^−1^)	2.66 ± 0.24	0.87 ± 0.02

a Hemagglutination activity was expressed as titer, the reciprocal of the highest two-fold dilution exhibiting positive hemagglutination.

The symbol “ - ” indicates no activity.

Microalgae contain substantially lower content of phytic acid (0.62 mg g^−1^) and trypsin inhibitor activity (0.52 mg g^−1^) than ESBM (Table 3). The content of phytic acid in microalgal biomass was only 3.6% of that in ESBM. Since the enzyme-based treatment effectively reduced the contents of ANFs in soybean meal, without any further treatment, the soybean meal may contain higher phytic acid and show greater trypsin inhibitor activity than microalgae. In contrast, the contents of total saponin (40.7 mg g^−1^) and tannins (2.7 mg g^−1^) in microalgal biomass were much higher than those in ESBM. Saponin contents in algae were positive without numerical data in most studies (42–47). Only Prabakaran et al. (48) reported that the saponin content of *Chlorella vulgaris* was 57.5 mg g^−1^, which was closed to our result (40.7 mg g^−1^). Saponins are a class of glycosides whose aglycones are triterpenoids or helical steranes, which have antibacterial, cholesterol-lowering and anticancer biological activities (49). However, saponins are toxic to cold-blooded animals because of their hemolysis (50). For instance, soy saponin as an ANF can cause metabolic disorders and hinder fish growth (51, 52). Such effect of fish growth may depend on the dosage of saponin in the daily dry diet. A low-dosage saponin (0.15 mg g^−1^ daily dry diet) could promote the growth and metabolic efficiency of fish (53–56). The addition of 1–2 mg g^−1^ saponins in the daily dry diet had no effect on the growth of fish (57–60). However, the addition of 10 mg g^−1^ saponins could reduce the growth and metabolic disorders of fish (52), and had the risk of inducing fish enteritis (61). In addition, saponin-containing diets negatively affect the taste acceptability and palatability of feed (62, 63). Therefore, if microalgal biomass is applied to the fish diet to replace fishmeal or ESBM, the contents of total saponin and tannins should be reduced. Otherwise, these ANFs may pose a threat to the performance and health of aquatic animals.

### Molecular characterization of proteins

In aquaculture, the molecular weight of proteins in fish diet is an important concern. In most cases, proteins with high molecular weights may not be efficiently digested by animals (64). Therefore, proteins with lower molecular weights are preferred in the preparation of a fish diet. For example, ESBM is a soybean meal treated by several enzymes which can convert proteins with high-molecular weights to low-molecular weights.

**Figure 1:**
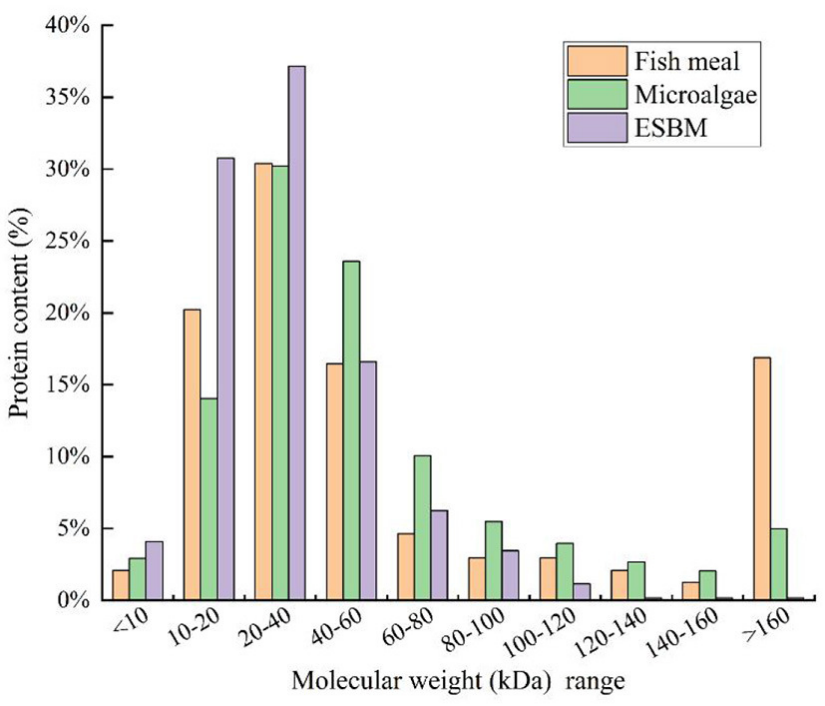
Molecular weight distributions and corresponding protein contents of fishmeal, microalgae, and enzyme-treated soybean meal (ESBM).

As shown in Figure 1, over 30% and 35% of proteins in ESBM ranged from 10–20 to 20–40 kDa, respectively. Hence, the majority of proteins in ESBM were in the form of low-molecular-weight components. Compared to ESBM, microalgae contained high-molecular-weight proteins. Figure 1 shows that nearly 13% and 30% of proteins in microalgal biomass ranged from 10–20 to 20–40 kDa, respectively. Additionally, about 23% of proteins in microalgal biomass ranged from 40 to 60 kDa. Although both microalgae and ESBM are plant-based protein sources, their protein molecular distributions are different. Therefore, pretreatment may be adopted to lower the molecular weights of proteins before the addition of microalgae to the fish diet.

## Discussion

### Alternative nutrition source

In aquaculture, to promote the fast growth of fish, sufficient nutrients, particularly proteins, should be supplemented (65). In the past, fishmeal was regarded as a good protein source for aquatic animals. However, with the depletion of fisheries in some regions, alternative nutrition source is gaining popularity (2).

To lower the total cost of the fish diet, plant-based proteins are a cost-saving feed ingredient. ESBM has been utilized to partly replace fishmeal for the production of fish feed (66). In practice, intensive utilization of soybean meal in aquaculture may cause an insufficient supply of soybean products, resulting in food security problems. The widespread cultivation of soybean may consume a huge chunk of land resources, disturbing the cultivation of other crops (67). Since microalgae production poses no conflict with traditional agriculture, microalgal biomass enriched with proteins is a potential alternative nutrition source for aquaculture.

### Production of microalgae-based fish diet

In this study, interesting discoveries, which are of importance to the practical application of microalgae in fish diet, were reported. First, compared to fishmeal, microalgae biomass lacks some essential nutrients and elements, like Ca. Moreover, the protein content in microalgae was lower than that in fishmeal. Hence, in practice, to substitute fishmeal with microalgae, additional nutrients should be added to the fish diet to improve its nutritional value. Second, microalgae contained some bioactive components, which might be sensitive to heating treatment. In the industry, to produce a microalgae-based fish diet, the temperature should be strictly regulated, else the nutritional values of microalgae may be severely reduced. Third, we found some ANFs in microalgae, which could negatively impact fish productivity. Therefore, pretreatment is necessitated to reduce the contents of ANFs in microalgae. Finally, measures should be taken to further reduce the molecular weights of proteins in microalgae to promote their digestion in fish culture.

## Data availability statement

The original contributions presented in the study are included in the article/Supplementary material, further inquiries can be directed to the corresponding authors.

## Author contributions

FC and YL: analysis, writing and revision. YH: revision. JQ and WZ: supervision. All authors contributed to the article and approved the submitted version.
