# Supplementary material for: Could Chlorella pyrenoidosa be exploited as an alternative nutrition source in aquaculture feed? A study on the nutritional values and anti-nutritional factors
Source: Front Nutr. 2022 Dec 7;9:1069760. doi: 10.3389/fnut.2022.1069760 (PMC9768438; doi:10.3389/fnut.2022.1069760)
Supplement: Supplementary file 1 [file Table_1.DOCX]

# Table S1

# Elemental analysis of fishmeal, microalgae, and enzyme-treated soybean meal (ESBM)

|  | Fishmeal | Microalgae | ESBM |
| --- | --- | --- | --- |
| Phosphorus (%) | 3.65 ± 0.26 | 2.09 ± 0.11 | 1.24 ± 0.14 |
| Calcium (%) | 4.73 ± 0.12 | 0.28 ± 0.02 | 0.18 ± 0.01 |
| Ca/P | 1.30 | 0.14 | 0.15 |
| Magnesium (%) | 0.22 ± 0.01 | 0.36 ± 0.02 | 0.14 ± 0.01 |
| Potassium (%) | 1.13 ± 0.08 | 1.25 ± 0.07 | 1.56 ± 0.09 |
| Sodium (%) | 1.32 ± 0.01 | 0.81 ± 0.05 | 0.09 ± 0.01 |

# Table S2

# Fatty acid composition of fishmeal, microalgae, and enzyme-treated soybean meal (ESBM)

| Fatty Acid (FA)  (%) | Fishmeal | Microalgae | ESBM |
| --- | --- | --- | --- |
| Long chain fatty acids | 81.55 | 76.22 | 41.11 |
| Saturated fatty acid | 37.13 | 29.94 | 24.8 |
| Unsaturated fatty acid | 44.42 | 46.28 | 16.31 |
| Monounsaturated fatty acids | 22.61 | 45.09 | 9.31 |
| Polyunsaturated fatty acid | 21.81 | 1.19 | 7.00 |


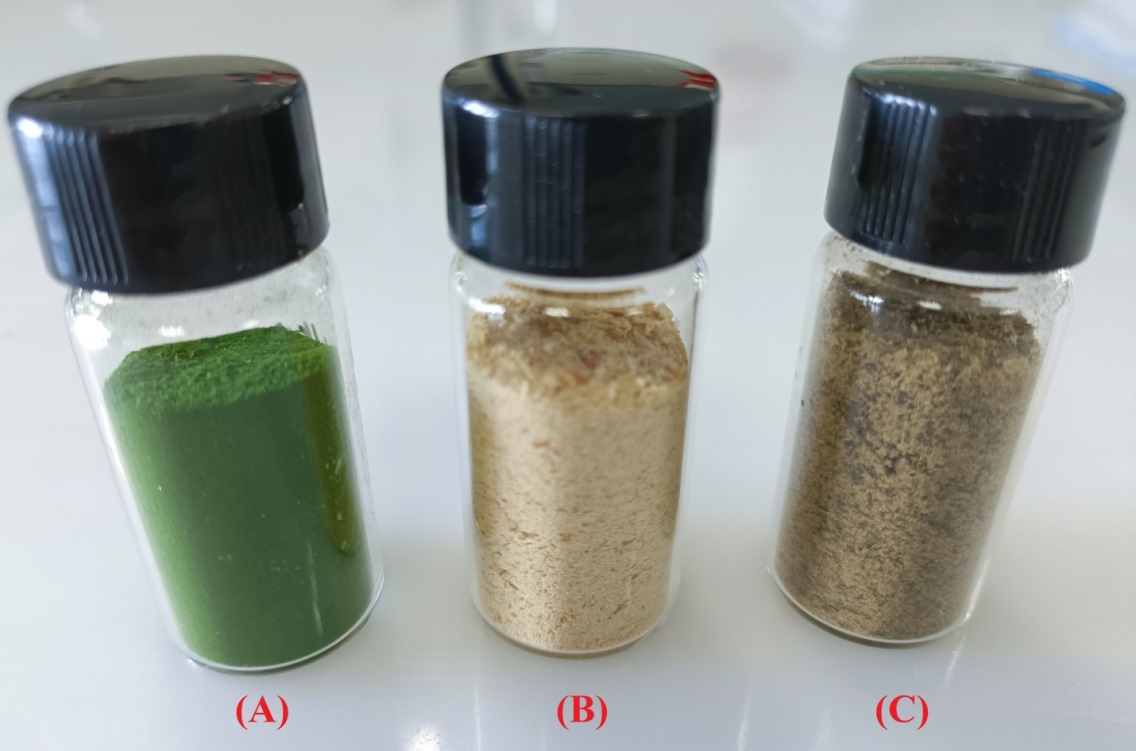


**Fig. S1.** Samples for experiment (A: Microalgal biomass; B: enzyme-treated soybean meal (ESBM); C: Fishmeal)
